# Idiopathic granulomatous mastitis associated with risperidone-induced hyperprolactinemia

**DOI:** 10.1186/1746-1596-7-2

**Published:** 2012-01-05

**Authors:** Chih-Hsun Lin, Chih-Wei Hsu, Tang-Yi Tsao, Jason Chou

**Affiliations:** 1 Antai Medical Care Cooperation Antai Tian-Sheng Memorial Hospital, Department of Surgery, Division of Plastic and Reconstructive Surgery, PingTung County, Taiwan; 2 Tungs' Taichung MetroHarbor Hospital, Department of Surgery, Division of Breast Surgery, Taichung, Taiwan; 3 Tungs' Taichung MetroHarbor Hospital, Department of Pathology, Taichung, Taiwan; 4 Cheng Hsin General Hospital, Department of Anatomic Pathology, Taipei, Taiwan

## Abstract

Idiopathic granulomatous mastitis (IGM) is a rare inflammatory breast disease. The etiology and treatment options of IGM remain controversial. Previous case reports have suggested that hyperprolactinemia may be associated with IGM. In the present report, we describe the first case of IGM associated with risperidone-induced hyperprolactinemia.

**Virtual slides:**

The virtual slide(s) for this article can be found here: http://www.diagnosticpathology.diagnomx.eu/vs/8120093785928228

## Background

Idiopathic granulomatous mastitis (IGM) is a rare benign breast disease characterized by non-caseating granulomas without any evidence of infection. This disease was first described by Kessler and Wolloch in 1972 [1]. It usually affects young parous women with a history of breast-feeding. The clinical presentation is usually a tender, erythematous breast mass with an abscess or chronic sinus drainage. However, the clinical and radiological features of this benign entity are frequently mistaken for malignancy. Several etiologies of IGM have been postulated, including immune reaction to extravasated milk secretion, breast trauma, or infection [2,3]. Definitive diagnosis is based on histological examination. Other causes of mammary granulomatous formation must be excluded prior to diagnosis, and microbiological investigation is necessary. Because of an absence of a consensus for an appropriate treatment modality for patients diagnosed with IGM, surgery, steroids, immunosuppressants, and antibiotics have been attempted with varying degrees of success. Recurrence is common in the absence of surgical treatment, and a long-term follow-up is generally essential.

Here we represent a case of granulomatous mastitis associated with risperidone-induced hyperprolactinemia. There have been only few cases associated with hyperprolactinemia, and this case could be another one belonging to this etiologic group.

## Case Presentation

**Figure 1:**
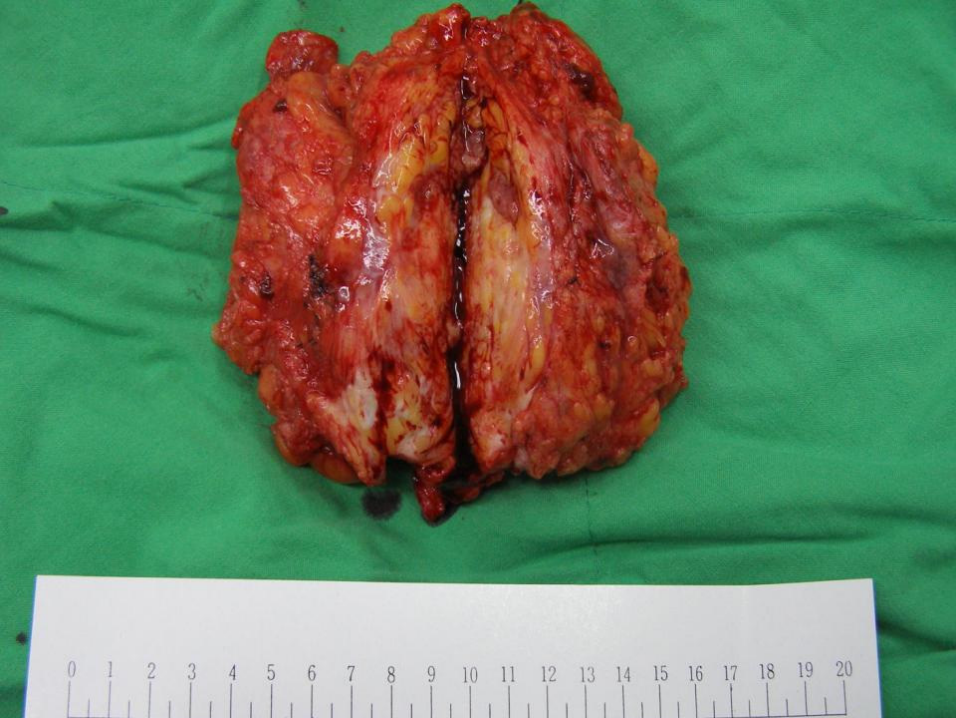
**A huge soft tissue mass (9 × 6 cm) with fibrosis, spotty necrosis, inflammation, and hemorrhage**. The virtual slides for this article can be found here: http://diagnosticpathology.slidepath.com/dih/webViewer.php?snapshotId=1320701553, http://diagnosticpathology.slidepath.com/dih/webViewer.php?snapshotId=1320701637, http://diagnosticpathology.slidepath.com/dih/webViewer.php?snapshotId=1320701673, http://diagnosticpathology.slidepath.com/dih/webViewer.php?snapshotId=1320701702.

**Figure 2:**
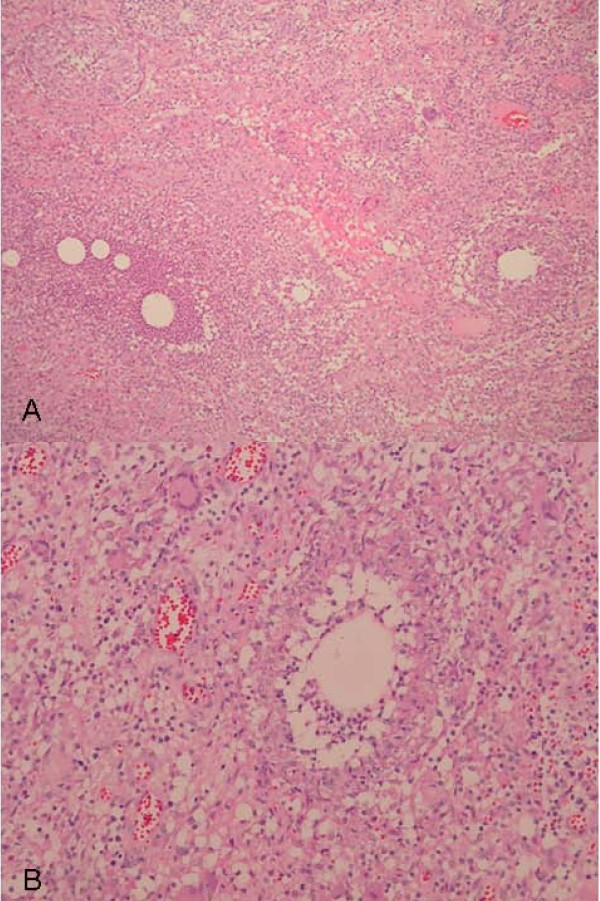
**Microscopic findings of idiopathic granulomatous mastitis**. 2A Empty spaces of varying sizes surrounded by granulomatous inflammation and micro-abscess formation (H & E, 40×) 2B Epithelioid granuloma admixed with polymorphonuclear cells and multinucleated giant cells. (H & E, 100×) The virtual slides for this article can be found here: http://diagnosticpathology.slidepath.com/dih/webViewer.php?snapshotId=1320701730, http://diagnosticpathology.slidepath.com/dih/webViewer.php?snapshotId=1320701765, http://diagnosticpathology.slidepath.com/dih/webViewer.php?snapshotId=1320701787.

The patient in this study was a 39-year-old Chinese woman who presented with a painful right breast lump on April 5, 2008. She had a history of schizophrenia for over 20 years and had been receiving risperidone (2 mg bid) for more than 3 years. She had developed a lump in her right breast a month before her visit and had noted a progressive worsening tenderness with erythematous changes around the lesion. She had no systemic symptoms nor did she have any known previous fungus or tuberculosis exposure. The patient had a gestational history of G0P0 and had amenorrhea for approximately a year. She had no history of any other breast disease or receiving any breast surgery, and her medical history was otherwise unremarkable. She had never consumed tobacco, alcohol, oral contraceptive pills nor did she have any family history of breast cancer. On physical examination, a 9-cm × 6-cm breast lump with localized redness over the right breast lateral aspect with an ill-defined margin was noted with no palpable lymphadenopathy at the axilla. There was no splenomegaly or hepatomegaly. She did not have fever, joint pain, airway or urinary tract bleeding. No other skin lesion was found. The patient's blood count was normal. Breast ultrasound images revealed a large ill-defined area with heterogeneous echoes in the right upper and lower outer quadrants, associated with increased vascularity. Micro-calcification and tissue edema were noted. No enlarged lymph node was noted in the right axilla, and there was no dilatation of the lactiferous duct. CXR didn't show any lung lesions. Mammography showed asymmetry with increasing radiodensity at the outer upper quadrant of the right breast with nipple retraction. Mastitis with edema and micro-abscess in the right upper and lower quadrants was suspected. However, inflammatory carcinoma could not be ruled out. All other laboratory and other radiological studies including C-reactive protein (0.8 mg/L) were normal except that the prolactin level was 84.5 ng/ml (normal, < 20 ng/ml); FSH, 4.6 mIU/ml; and LH, 6.0 mIU/ml. Although malignancy had not been excluded, the patient received a presumptive diagnosis of infectious mastitis and was treated with a 14-day course of keflex (first-generation cephalosporin), but there was no improvement. Therefore, core breast biopsy was performed, which revealed an adipose tissue with acute and chronic inflammation. Aspiration culture showed no evidence of bacterial or mycobacterial growth. In order to rule out previously missed diagnosis, another incision biopsy was performed 1 week later, and the pathology showed chronic inflammation with focal fibrotic changes in the fat tissues. Due to persistent drainage with erythematous swelling and in consideration of malignancy, the patient then was scheduled for simple mastectomy. Macroscopic appearance demonstrated a huge mass with an ill-defined margin of inflammatory tissue at the center with peripheral fatty necrosis and hematoma (Figure 1). Microscopic examination revealed chronic inflammation and macrophage, giant histiocyte, and epithelioid-like cellular infiltration, with cytologic features suggestive of a granulomatous process. The noncaseous granulomatous lesions were centered at the breast lobules. The lesions were characteristic with an empty space in the center and surrounded by microgranulomas and microabscesses. Some granulomatous lesions composed of confluent epithelioid cells and huge abscess were also seen (Figure 2A, B). Based on the histological features, the differential diagnosis included autoimmune response, undetected organisms, systemic granulomatous disease with breast involvement, granulomatous reaction in a carcinoma and foreign body reaction. There was no evidence of carcinoma or specific organism can be found. All cultures, Ziehl-Neelsen staining, PAS staining and GMS staining yielded negative results. Considering the patient's clinical history and all the laboratory findings, the diagnosis was interpreted as IGM. In suspect of the dopaminergic effect of risperidone to cause hyperprolactinemia and IGM, the prescription was shifted to prolactin-sparing second line agent (clozapine). The residual breast lesions resolved completely 3 months later and no recurrence was noted since then.

## Discussion

Idiopathic granulomatous mastitis is characterized by the presence of chronic granulomatous lobulitis. It is a disease of unknown etiology and is diagnosed by exclusion. Clinically, patients with IGM present a unilateral hard lump in the absence of any systemic signs of infection. These lumps mimic carcinoma and may lead to nipple retraction and sinus formation [1,2].

Approximately 200 cases of IGM have been reported in the literature during the past 3 decades, with most of them being reported in developing countries. More than 50% of the reported cases of IGM were initially mistaken for breast carcinoma, even with a combined diagnosis by mammography and ultrasound. A definitive diagnosis can be made only through histological evidence of granuloma formation with the absence of any infection. A well-formed granuloma centered at the terminal ducts and the lobules was noted, and this could occur anywhere in the breast. A mixed chronic inflammatory process composed of lymphocytes, plasma cells, epithelioid histiocytes, multinucleated giant cells, and a less frequent neutrophil infiltration with micro-abscess formation can be found in the granuloma [3,4].

Before diagnosing IGM, any known cause of granulomatous breast inflammation, such as duct ectasia, sarcoidosis, Wegener's granulomatosis, giant cell arteritis, polyarteritis nodosa, tuberculosis, syphilis, corynebacterial infection, cat-scratch disease in breast lymph node tissue, mycotic infection, granulomatous reaction in a carcinoma and foreign body reaction, whether infectious or non-infectious, should be ruled out [5-7]. Duct ectasia can mimic IGM as both possess similar clinical symptoms, including nipple retraction, pain, and occasional bloody discharge. The dilation of the large duct with peripheral duct inflammation, and not centered at the breast lobules, is histologically evident. Sarcoidosis is a systemic granulomatous disease of unknown etiology with high prevalence rate in European countries. It usually involves lung and associated lymph nodes and is histologically characterized by well-formed tight granulomas without necrosis, extensive inflammation, and abscesses. The breast is involved in less than 1% of cases [8]. Wegener's granulomatosis is a form of large-size vasculitis that usually affects upper airway, lung and kidney. It is featured as an autoimmune attack by an abnormal type of circulating antibody termed ANCAs (antineutrophil cytoplasmic antibodies). The histological presentation is small and medium vessel vasculitis with necrotizing granulomas. Breast involvement is rare and usually accompanied by systemic manifestations of the disease [9,10]. Polyarteritis nodosa and giant cell arteritis are occasionally classified as vasculitis, but actually are a form of arteritis since they mainly involve the arteries. Granulomatous response to carcinoma is unusual and characterized by multinucleated giant cells restricted to the carcinoma or osteoclastic giant cells (OGCs) at peripheral hypervascular stroma. These two types have been easily identified with intraductal or invasive carcinoma [11,12]. Foreign body reaction may be due to silicon leakage from breast implant. Although there are no risks of systemic disease or carcinogenesis, the relationship between silicon breast implant and primary anaplastic large cell lymphoma (ALCL) of the breast has been reported [13]. Culture specimens and special staining of specimens, including Ziehl-Neelsen staining, PAS staining, GMS staining, and Warthin-Starry silver impregnation, can rule out rare infectious granulomatous breast lesions.

The presumptive causes of IGM, including alpha-1-antitrypsin deficiency, oral contraceptives usage, pregnancy, lactation, and hyperprolactinemia have been reported [2,6,14,15]. Interestingly, hyperprolactinemia whether primary or secondary, might play an important role in IGM. It has been postulated that IGM results from a localized autoimmune response to the retained or extravasated fat- or protein-rich secretion in the breast ducts during childbearing age due to a previous hyperprolactinemia in women [16]. To the contrary, as the occurrence of IGM without a history of recent pregnancy is uncommon, the serum prolactin level and the pathogenesis of IGM in non-pregnant women therefore might help us understand the role of hyperprolactinemia in the disease. Especially, the role of hormone in mammary tumor growth is under investigated and with some trigger agents, such as environmental contaminants, the increase of hormone receptors can promote cell proliferation [17]. To the best of our knowledge, IGM in non-pregnant women associated with high serum prolactin level can has been rarely reported associated with pituitary adenoma, phenothiazine-induced hyperprolactinemia, and metoclopramide-related galactorrhea with blunt trauma [16,18-20]. Our case is the fourth one in a non-pregnant woman with hyperprolactinemia and is the first case to be reported that the hyperprolactinemia was induced by risperidone.

Many antipsychotic agents are known to increase prolactin secretion because of their inhibitory effect of dopamine. Risperidone is known to increase serum prolactin level to a greater extent than other antipsychotics, such as quetiapine. It is thought that once the prolactin level is elevated due to risperidone, it causes non-cancerous tumors in the pituitary gland. This has been demonstrated to recur even after the drug has been switched to a different class of antipsychotic agents [21,22]. Interestingly, over the past decades, several studies have specifically examined the relationship between prolactin levels and granulomatous diseases. These studies suggest that prolactin contributes to a wide variety of both physiological and pathological granulomatous cutaneous lesions, especially those of immune response, such as the non-caseating granulomas [23,24]. The clinical presentation of IGM patients is similar, despite the varied causes behind the laboratory evidence of hyperprolactinemia or galactorrhea. Based on these findings, hyperprolactinemia and/or galactorrhea along with other superimposing causes, such as local trauma or irritation may be the sequential features that result in IGM.

There is no consensus regarding the ideal management of IGM. A conservative non-operative treatment is usually recommended for IGM patients with mild symptoms [25]. Therapy is recommended for patients with drug-induced hyperprolactinemia, and these patients are usually shifted to a prolactin-sparing agent. For patients with more severe symptoms, oral prednisolone is administered [26]. Immunosuppressants may be effective in refractory cases of the above regimens [27,28]. Finally, in persistent or recurrent cases, wide surgical excision and/or mastectomy should be considered [2,29].

## Conclusions

Idiopathic granulomatous mastitis is an uncommon benign entity of the breast with a diagnosis exclusive of malignancy and infectious etiology. In this study, we report a case with this mysterious entity in a non-pregnant woman with a laboratory evidence of hyperprolactinemia due to risperidone usage. Investigating more number of case reports and further assessments are necessary for understanding the relationship between hyperprolactinemia and IGM.

## Consent

Written informed consent was obtained from the patient for publication of this Case Report and any accompanying images. A copy of the written consent is available for review by the Editor-in-Chief of this journal.

## Competing interests

The authors declare that they have no competing interests.

## Author's information

^1 ^No.210, Sec. 1, Zhongzheng Rd., Donggang Township, Pingtung County 928, Taiwan. ^2,3 ^No.699, Sec. 1, Zhongqi Rd., Wuqi Dist., Taichung City 435, Taiwan. ^4 ^No.45, Cheng Hsin St., Pai-Tou, Taipei.

## Authors' contributions

CH-L constructed the majority of the manuscript and CW-H was responsible for the surgical consultation. JC and TY-T provided pathological assistance and were responsible for a significant portion of the manuscript. All authors read and approved the final manuscript.
